# Sports therapy interventions and symptom severity in unipolar depression: an intervention study

**DOI:** 10.1192/j.eurpsy.2025.676

**Published:** 2025-08-26

**Authors:** C. A. Penkov, M. Wendt, K. Friedrich, C. Linke, L. Duda, M. Ziegenbein

**Affiliations:** 1 Klinikum Wahrendorff, 31319, Germany

## Abstract

**Introduction:**

Physical activity can be therapeutically effective in the treatment of depressive disorders. There is a need for research in clinical practice both with regard to the framework conditions required for physical activity and whether the implementation of sports therapy under the conditions of everyday care can have an effect on physical fitness and depressive symptoms. This study therefore examines the effect of sports and exercise therapy in the day-care treatment of unipolar depression.

**Objectives:**

- treatment of depressive disorders
- implementation of sports therapy under the conditions of everyday care

**Methods:**

Patients with a depressive disorder as their main clinical diagnosis (F32./F33.) who underwent day clinic treatment for 5-11 weeks were included. People in the intervention and control groups completed a minimum of 2.0 and a maximum of 0.5 exercise sessions per week respectively. To investigate the effect of sports and exercise therapy on aerobic performance, the intervention group completed a submaximal, bicycle ergometric step test (PWC test), whereby the heart rate was measured over the individual exercise levels in a pre-post comparison. Furthermore, the change in depression symptoms between the intervention and control group was recorded at admission and discharge from treatment using the BDI-II.

**Results:**

Patients in the intervention group (IG) showed a significantly greater reduction in depression symptoms compared to the control group (CG) (ΔBDI-II; M = - 8, p <.01).

In the pre-post comparison of PWC, IG achieved a significant increase in performance of 7 and 12 watts respectively (p <.05; t-test). Further inferential statistical results are reported.

**Conclusions:**

Regular physical training can lead to a significant improvement in endurance performance and an improvement in depressive symptoms.

**Disclosure of Interest:**

None Declared

